# Cost-Utility Analysis of Camrelizumab Plus Chemotherapy Versus Chemotherapy Alone as a First-Line Treatment for Advanced Nonsquamous Non-Small Cell Lung Cancer in China

**DOI:** 10.3389/fonc.2022.746526

**Published:** 2022-07-22

**Authors:** Ting Chen, Ruixiang Xie, Qiuling Zhao, Hongfu Cai, Lin Yang

**Affiliations:** ^1^ Department of Pharmacy, Fujian Medical University Cancer Hospital, Fujian Cancer Hospital, Fuzhou, China; ^2^ Department of Pharmacy, Fujian Medical University Union Hospital, Fuzhou, China

**Keywords:** nonsquamous NSCLC, camrelizumab, chemotherapy, first-line treatment, cost-utility analysis

## Abstract

**Purpose:**

To evaluate the cost utility of camrelizumab plus standard chemotherapy versus standard chemotherapy alone as a first-line treatment for advanced nonsquamous non-small cell lung cancer (NSCLC) from the perspective of the Chinese health care system and to provide a reference for health decision-making.

**Methods:**

A Markov model consisting of three health states was designed to evaluate the cost utility of these two treatment regimens for NSCLC patients with the incremental cost-effectiveness ratio (ICER) as the primary output indicator. Clinical data were derived from a published phase III clinical trial (CameL; ClinicalTrials.gov; NCT03134872). One-way sensitivity analysis and probabilistic sensitivity analysis were performed to assess the model uncertainty.

**Results:**

Base case analysis showed that the ICER of camrelizumab plus chemotherapy compared with chemotherapy alone was $43,275.43 per QALY. It was higher than the willingness-to-pay (WTP) threshold of $31,510.57 per QALY in China, which has a standard of three times the GDP per capita recommended by the WHO. One-way sensitivity analysis showed that the utility value of PFS had the greatest influence on the results, and the other sensitive parameters were the cost of subsequent second-line therapy in the two group, the pemetrexed price, the cost of adverse event management and the utility value of PD. The probability sensitivity analysis showed that the probabilities of the cost-effectiveness of camrelizumab plus standard chemotherapy were 27.1%, 66.7% and 88.0% when the WTP values were $40,000, $50,000 and $60,000 per QALY, respectively.

**Conclusions:**

Taking three times the GDP per capita in China as the WTP threshold, the camrelizumab plus standard chemotherapy regimen does not have a cost-effectiveness advantage compared with the standard chemotherapy regimen alone as a first-line treatment for advanced NSCLC.

## Introduction

Lung cancer is a kind of malignant tumor that seriously endangers human health, ranking first in incidence and mortality among malignant tumors in China (1). Non-small cell lung cancer (NSCLC) is the most common type of lung cancer, accounting for 85% to 90% of cases in China (2, 3). Most cases of lung cancer are found in an advanced stage when they are first diagnosed, and they lose the opportunity for surgery. From 2012 to 2014, the proportion of stage IIIA-IV lung cancer in China was 64.6% (2, 3). Over the past decade, the treatment of NSCLC has focused on systemic chemotherapy to extend the survival and improve the quality of life of advanced-stage patients. For example, based on histology, pemetrexed combined with platinum-based chemotherapy and maintenance therapy with pemetrexed is often used. With the development of molecular biology, lung cancer has ushered in the era of targeted therapy. Moreover, the development of immune checkpoint inhibitors (ICIs) in recent years has become a new milestone in cancer treatment, allowing untreated or multiline NSCLC patients to benefit from this treatment (4).

As a humanized immunoglobulin G4 monoclonal antibody, camrelizumab binds to the programmed cell death 1 (PD-1) receptor, blocks the binding of PD-1 and its ligand to reactivate T cells, and plays an antitumor role (5–8). Based on the results of CameL (9), camrelizumab has been approved in addition to pemetrexed and carboplatin as a first-line treatment for advanced or metastatic nonsquamous NSCLC and has been included in the Chinese Society of Clinical Oncology NSCLC guidelines for first-line treatment recommendations. The CameL study showed that the median overall survival (mOS) of camrelizumab plus chemotherapy as first-line treatment for advanced nonsquamous NSCLC was 27.9 months. Although the success of breaking through the two-year mark will bring good survival benefits to patients, high prices may bring a heavy socioeconomic burden to patients and the healthcare system. There is no uniform conclusion about its economic impact at present (10, 11), and the systematic review showed that checkpoint inhibitors as a first-line treatment for NSCLC has no economic advantage versus conventional chemotherapy regardless of PD-L1 level (12). Therefore, a cost-utility evaluation was performed to compare camrelizumab plus standard chemotherapy with standard chemotherapy alone as a first-line treatment for advanced nonsquamous NSCLC from the perspective of the Chinese health care system in this study. Information on these factors is required by healthcare decision makers to choose more reasonable treatments (13, 14).

## Materials and Methods

### Clinical Data

Data were derived from CameL (9), which was a randomized, open-label, multicenter, phase III clinical trial conducted in 52 hospitals in China. The inclusion criteria were as follows: patients histologically or cytologically diagnosed with stage IIIB-V NSCLC, with measurable lesions, without prior systemic antitumor therapy, without epidermal growth factor receptor (EGFR) and anaplastic lymphoma kinase (ALK) alterations, without active brain metastasis, and with an Eastern Cooperative Oncology Group (ECOG) performance status of 0 or 1. A total of 412 patients were randomly divided (1:1) into a camrelizumab (200 mg) plus pemetrexed (500 mg/m^2^) with carboplatin (area under curve [AUC], 5 mg/mL per min) group (n=205) or a pemetrexed (500 mg/m^2^) with carboplatin (AUC 5 mg/mL per min) group (n=207), with a treatment cycle every 21 days. Patients in the two groups received 4-6 cycles of the above treatment and then maintenance therapy. The total camrelizumab exposure was up to a maximum of 34 cycles. The median progression-free survival (mPFS) was 11.3 months vs 8.3 months (hazard ratio [HR] =0.6, 95% confidence interval [CI]: 0.45-0.79, P =0.0001), and the mOS was 27.9 months vs 20.5 months in the camrelizumab plus chemotherapy group vs the chemotherapy alone group (HR=0.73, 95% CI: 0.55-0.96, P = 0.0117). Severe treatment-related adverse events occurred in 74 (36%) patients in the camrelizumab plus chemotherapy group and in 27 (13%) patients in the chemotherapy alone group.

### Model Structure

**Figure 1:**
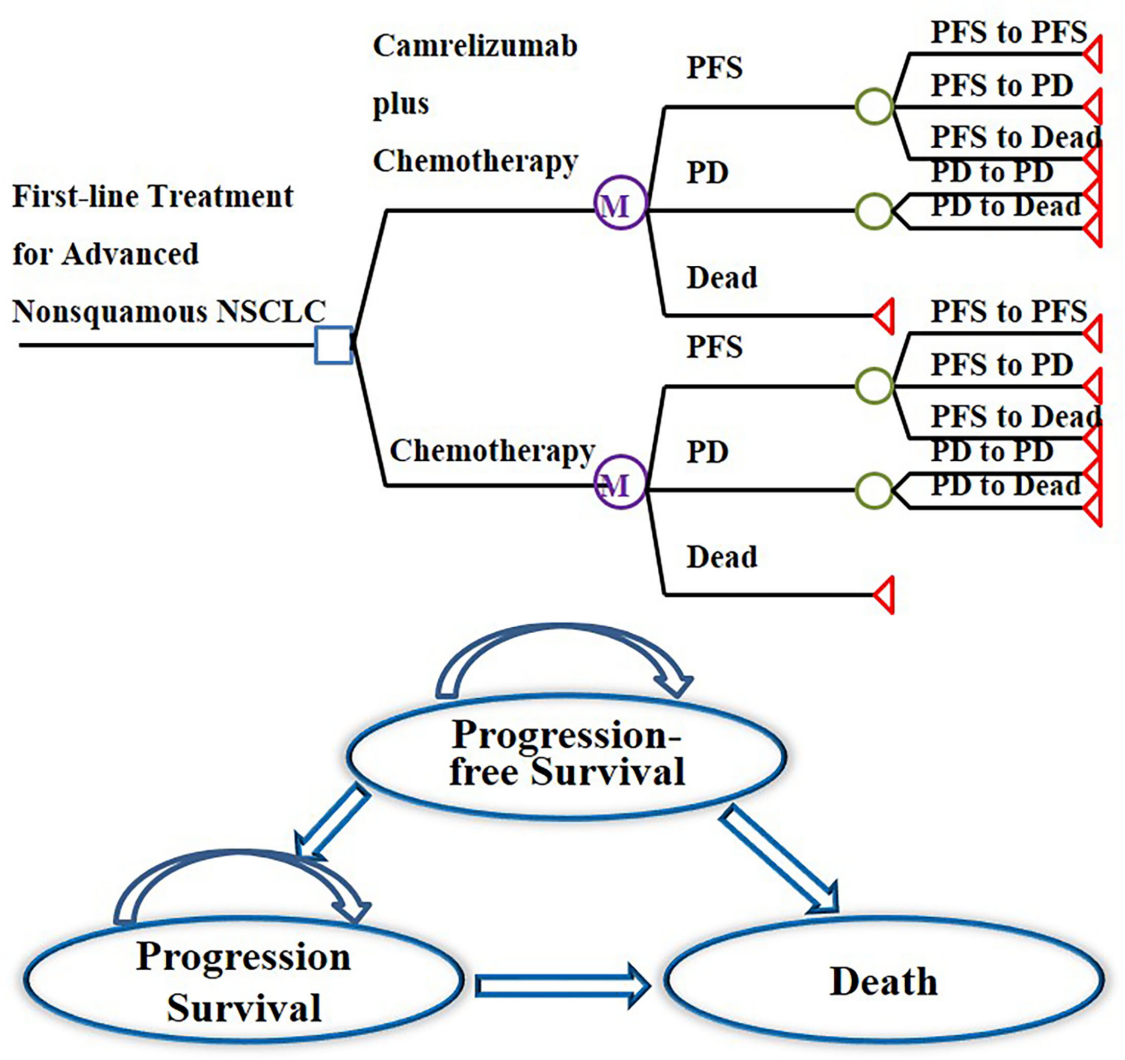
Markov model structure of camrelizumab plus chemotherapy versus chemotherapy alone as first-line treatment for advanced nonsquamous NSCLC and bubble diagram for NSCLC.

In this study, cost-utility analysis was carried out by constructing and simulating a Markov model run in TreeAge Pro 2011 (TreeAge Software Inc, Williamstown, MA, USA). According to the progression of the tumor, the structure of the model was composed of three independent states: PFS state, progression survival state, and death. All patients were in a progression-free state when they were enrolled, and the death state was an absorbed state, as shown in **Figure 1**. The cycle period of the model was 21 days. The model simulation showed that almost all patients in the two groups died after 10 years. Therefore, the time limit of this study was set at 10 years (15–17). The output indicators of the model were the cost and quality-adjusted life years (QALYs) of the two groups, and then the incremental cost-effectiveness ratio (ICER) was calculated.

### Transition Probability

Engauge Digitizer software (version 12.1, https://github.com/markummitchell/engauge-digitizer/releases) was used to extract the PFS and OS curve data from the CameL study, a Weibull distribution was used to fit and extrapolate the Kaplan-Meier (KM) curves of the two groups, and R software (version 4.1.0, https://cran.r-project.org/mirrors.html) was used to obtain the scale parameter (λ) and shape parameter (γ) of the function, as shown in **Table 1**. The transition probability was calculated according to the principle of DEALE (p = 1 – *e* ^–^ *^rt^*) (18). We assumed that the probability of transitioning from the PFS state to death was the local natural death rate.

**Table 1:** Parameters for Weibull survival curve fitting.

Survival Curve-Fitting	Scale (λ)	Shape (γ)
Survival Curve Fitting of PFS of Camrelizumab plus Chemotherapy	0.021350	1.210605
Survival Curve Fitting of OS of Camrelizumab plus Chemotherapy	0.006578	1.263333
Survival Curve Fitting of PFS of Chemotherapy	0.057642	1.019433
Survival Curve Fitting of OS of Chemotherapy	0.010970	1.216254

### Utility

Since the CameL study did not collect utility data, this study quoted the utility value of Chinese patients with metastatic nonsquamous NSCLC from published literature. The utility of PFS was 0.804, and the utility of progressive disease (PD) was 0.321 (19, 20).

### Cost Inputs

From the perspective of the Chinese health care system, this study considered only direct medical costs, including drug costs, drug management costs, disease management costs, and the cost of handling adverse events of level 3 and above. The adverse event processing cost was equal to the incidence of grade 3 and above adverse reactions times the processing cost of a single event. **Table 2** shows the grade 3 or more treatment-related adverse events in CameL with an incidence greater than 3% (9). Reactive cutaneous capillary hyperplasia (RCCEP) was the most common immune-related adverse event of camrelizumab, but most of the cases were grade 1, with only 2 cases of grade 3 (<1%) and no grade 4-5 adverse events. The incidence was reported; therefore, the cost calculation was not included in this article.

**Table 2:** Incidence rate of TRAEs.

	Camrelizumab plus Chemotherapy (n = 205)	Chemotherapy (n = 207)
Neutrophil count decreased	78 (38%)	63 (30%)
White blood cell count decreased	40 (20%)	30 (14%)
Anemia	38 (19%)	23 (11%)
Platelet count decreased	34 (17%)	24 (12%)
Lymphocyte count decreased	8 (4%)	5 (2%)
Alanine aminotransferase increased	10 (5%)	5 (2%)
Asthenia	7 (3%)	3 (1%)
Gamma-glutamyltransferase increased	6 (3%)	1 (<1%)

The price of the drug in this study is the median value of the bid-winning price of each province (21), and other costs come from the charging standard of a tertiary hospital in Fujian Province in China. The price of camrelizumab is the latest price after medical insurance negotiations in 2020. Based on the actual situation, there is no room for further increase, so only the impact of the decline in the price of camrelizumab on the results is considered. According to the “Guidelines for the Evaluation of Chinese Pharmacoeconomics (2020)”, all costs and output are discounted at an annual discount rate of 5% (22). The average exchange rate of Chinese Yuan renminbi for the entire year of 2020 is 6.8974 yuan per US dollar. It is assumed that the corresponding cost is incurred when the treatment is started in each cycle; therefore, the cost is not adjusted for the half cycle. The average body surface area is 1.71 m^2^, which was calculated from the average height and weight data of Chinese adults obtained in the “Report on Nutrition and Chronic Disease Status of Chinese Residents (2020)” (23) and the sex ratio of patients in the CameL study according to Xu Wensheng’s formula. The dose range and the lower limit of the treatment course were selected for the drug dosage and course of treatment. Referring to the other studies, it is assumed that docetaxel was used as the subsequent second-line chemotherapy, and nivolumab and camrelizumab as the immunotherapy, which is commonly used in China (10). The drug management cost was equal to the hospitalization cost plus the chemotherapy drug preparation injection cost. The two groups of patients were hospitalized for 3 days per cycle, the hospitalization cost per cycle was $55.67, and the chemotherapy drug preparation injection cost was different. The cost of drug administration in the chemotherapy alone group in the PFS state (>4 cycles) was the same as the cost of drug administration in the camrelizumab plus chemotherapy group in the PFS state (>34 cycles) (24, 25). The cost details are shown in **Table 3**.

**Table 3:** Model parameters and distribution.

Parameters	Base value	Low	High	Distribution	Source
Cost					
Cost of camrelizumab, US$	424.51	318.38	424.51	Gamma	(21)
Cost of pemetrexed, US$	393.99	187.78	402.11	Gamma	(21)
Cost of carboplatin, US$	11.96	3.84	22.91	Gamma	(21)
Cost of chemotherapy pretreatment, US$	1.52	1.14	1.89	Gamma	Local charge
Cost of subsequent second-line therapy in the camrelizumab plus chemotherapy group, US$	392.61	294.46	490.76	Gamma	(10)
Cost of subsequent second-line therapy in the chemotherapy alone group, US$	698.77	524.08	873.46	Gamma	(10)
Cost of drug administration in the camrelizumab plus chemotherapy group in the progression-free survival state (≤4 cycles), US$	72.54	54.40	90.67	Gamma	Local charge
Cost of drug administration in the camrelizumab plus chemotherapy group in the progression-free survival state (>4 cycles and ≤34 cycles), US$	66.27	49.70	82.84	Gamma	Local charge
Cost of drug administration in the camrelizumab plus chemotherapy group in the progression-free survival state (>34 cycles), US$	65.58	49.19	81.98	Gamma	Local charge
Cost of drug administration in the chemotherapy alone group in the progression-free survival state (≤4 cycles), US$	71.85	53.89	89.82	Gamma	Local charge
Cost of drug administration in the chemotherapy alone group in the progression survival state (≤34 cycles), US$	56.36	42.27	70.45	Gamma	Local charge
Cost of disease management in the first 18 cycles, US$	60.64	45.48	75.80	Gamma	Local charge
Cost of disease management after 18 cycles, US$	48.68	36.51	60.85	Gamma	Local charge
Cost of adverse reactions in the camrelizumab plus chemotherapy group, US$	120.06	90.05	150.08	Gamma	Local charge
Cost of adverse reactions in the chemotherapy alone group, US$	80.32	60.24	100.40	Gamma	Local charge
Utility values					
Utility of PFS	0.804	0.536	0.883	Beta	(19, 20)
Utility of PD	0.321	0.05	0.473	Beta	(19, 20)
Discount rate (%)	5	0	8		(22)

### Sensitivity Analysis

One-way sensitivity and probabilistic sensitivity analyses were used to evaluate the stability of the model results. One-way sensitivity analysis is used to analyze the influence of different parameters on ICERs in a certain range, and the results are presented by a tornado diagram. When the value range of the parameter can not be obtained, the variation range is ±25%. Considering the actual situation, the price of camrelizumab does not have the possibility of increasing, so only the impact of the price decline on ICER was analyzed. The probabilistic sensitivity analysis was performed using a bivalent Monte Carlo simulation. Assuming that each parameter follows a certain probability distribution, 1,000 repeated samples were taken, and ICER values of different treatment regimens were calculated based on each sample. The results of probabilistic sensitivity analysis are presented as an incremental cost-effectiveness scatter plot and an acceptability curve (26–28).

## Results

### Base Case Analysis

The analysis results of the Markov model established in this study showed that within the 10-year study period, both camrelizumab plus chemotherapy and first-line chemotherapy alone had clinical benefits in the treatment of advanced NSCLC. Compared with chemotherapy alone, camrelizumab plus chemotherapy could add 0.34 QALYs, with an incremental cost of $14,818.05 and a calculated ICER of $43,275.43 per QALY, as shown in **Table 4**. In 2020, the Chinese per capita GDP was $10,503.52 (29), so the willingness-to-pay (WTP), which is three times the per capita GDP according to the WHO, was $31,510.57. The ICER value was higher than the WTP, indicating that chemotherapy combined with camrelizumab was not economical compared with chemotherapy alone.

**Table 4:** Basic analysis results.

	Costs, US$	ΔCosts, US$	QALYs	ΔQALYs	ICER, US$/QALY
Chemotherapy	30,575.23	0	1.10	0	0
Camrelizumab plus Chemotherapy	45,393.28	14,818.05	1.44	0.34	43,275.43

### Sensitivity Analysis

#### One-Way Sensitivity Analysis

**Figure 2:**
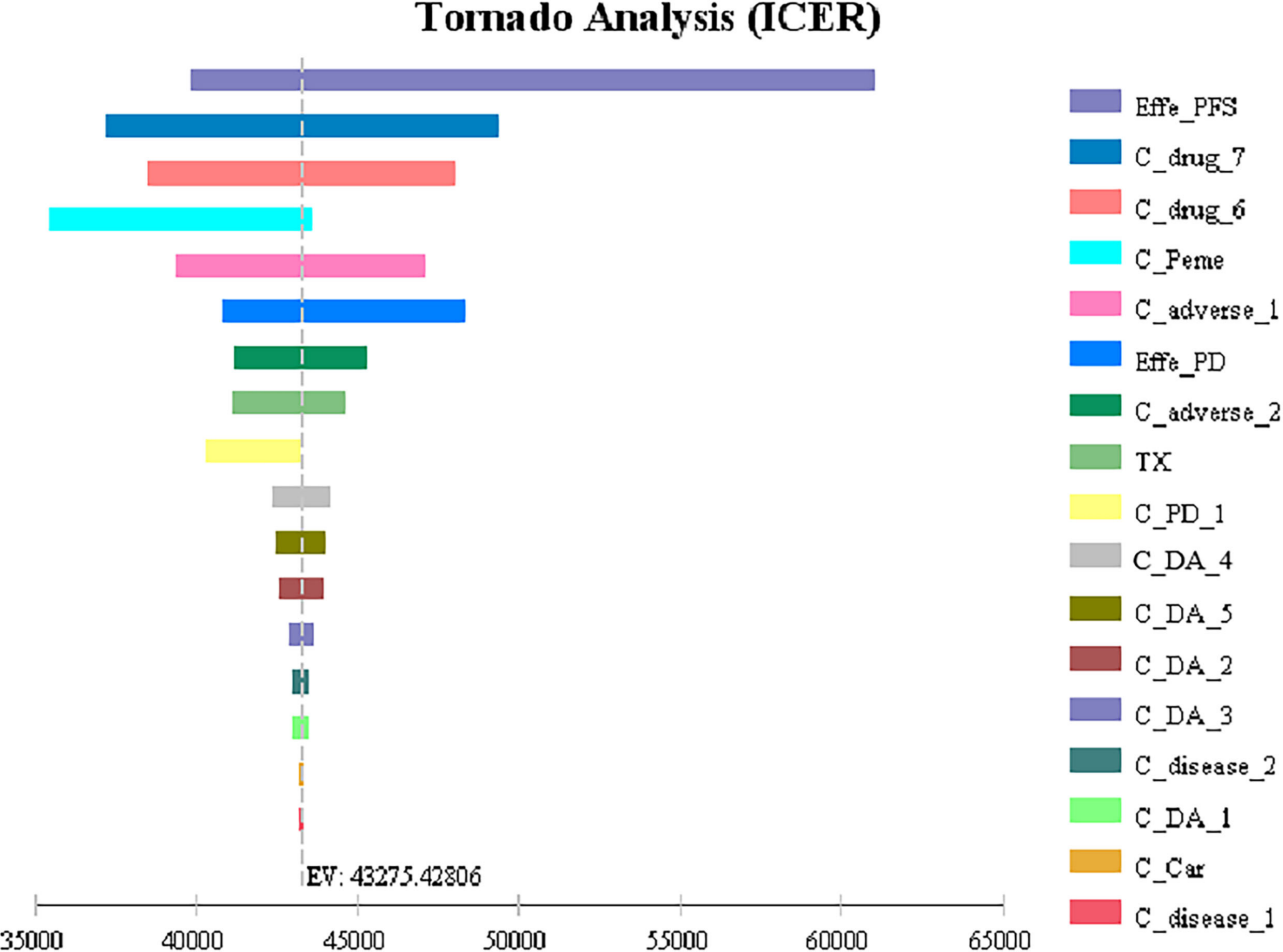
Tornado diagrams for one-way sensitivity analysis Effe_PD, utility of PD; Effe_PFS, utility of PFS; C_Peme, cost of pemetrexed; C_drug_6, cost of subsequent second-line therapy in the chemotherapy group; C _drug_7, cost of subsequent second-line therapy in the camrelizumab plus chemotherapy group; C_adverse_2, cost of adverse reactions in the chemotherapy alone group; C_adverse_1, cost of adverse reactions in the camrelizumab plus chemotherapy group; C_PD_1, cost of the PD-1 inhibitor camrelizumab; TX, discount rate (%); C_Car, cost of carboplatin; C_disease_1, cost of disease management in the first 18 cycles; C_disease_2, cost of disease management after 18 cycles; C_pretreat, cost of chemotherapy pretreatment; C_DA_5, cost of drug administration in the chemotherapy alone group in the progression survival state (≤34 cycles); C_DA_4, cost of drug administration in the chemotherapy alone group in the progression-free survival state (≤4 cycles); C_DA_3, cost of drug administration in the camrelizumab plus chemotherapy group in the progression-free survival state (>34 cycles); C_DA_2, cost of drug administration in the camrelizumab plus chemotherapy group in the progression-free survival state (>4 cycles and ≤34 cycles); C_DA_1, cost of drug administration in the camrelizumab plus chemotherapy group in the progression-free survival state (≤4 cycles).

The results of one-way sensitivity analysis showed that the utility of PFS had the greatest influence on the results, and the other sensitive parameters were the cost of subsequent second-line therapy in the two group, the pemetrexed price, the cost of adverse event management and the utility value of PD. However, ICER generated when all parameters change within a certain range does not intersect with WTP, so the model is robust. The analysis results are shown in **Figure 2**.

#### Probabilistic Sensitivity Analysis

**Figure 3:**
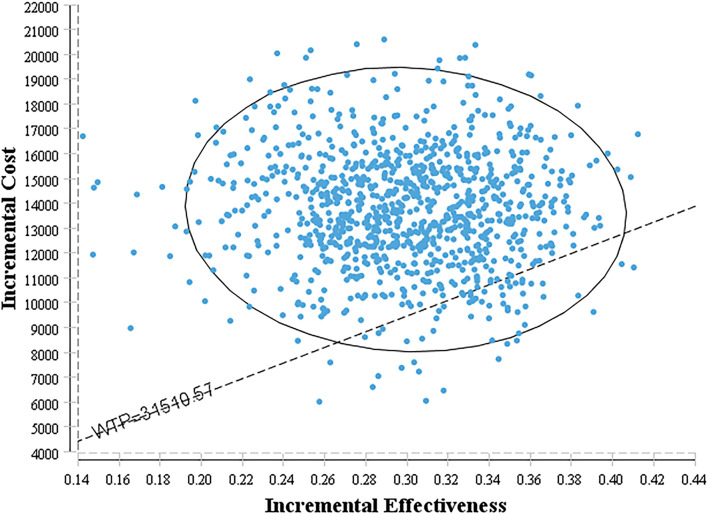
Cost-effectiveness scatter plot.

**Figure 4:**
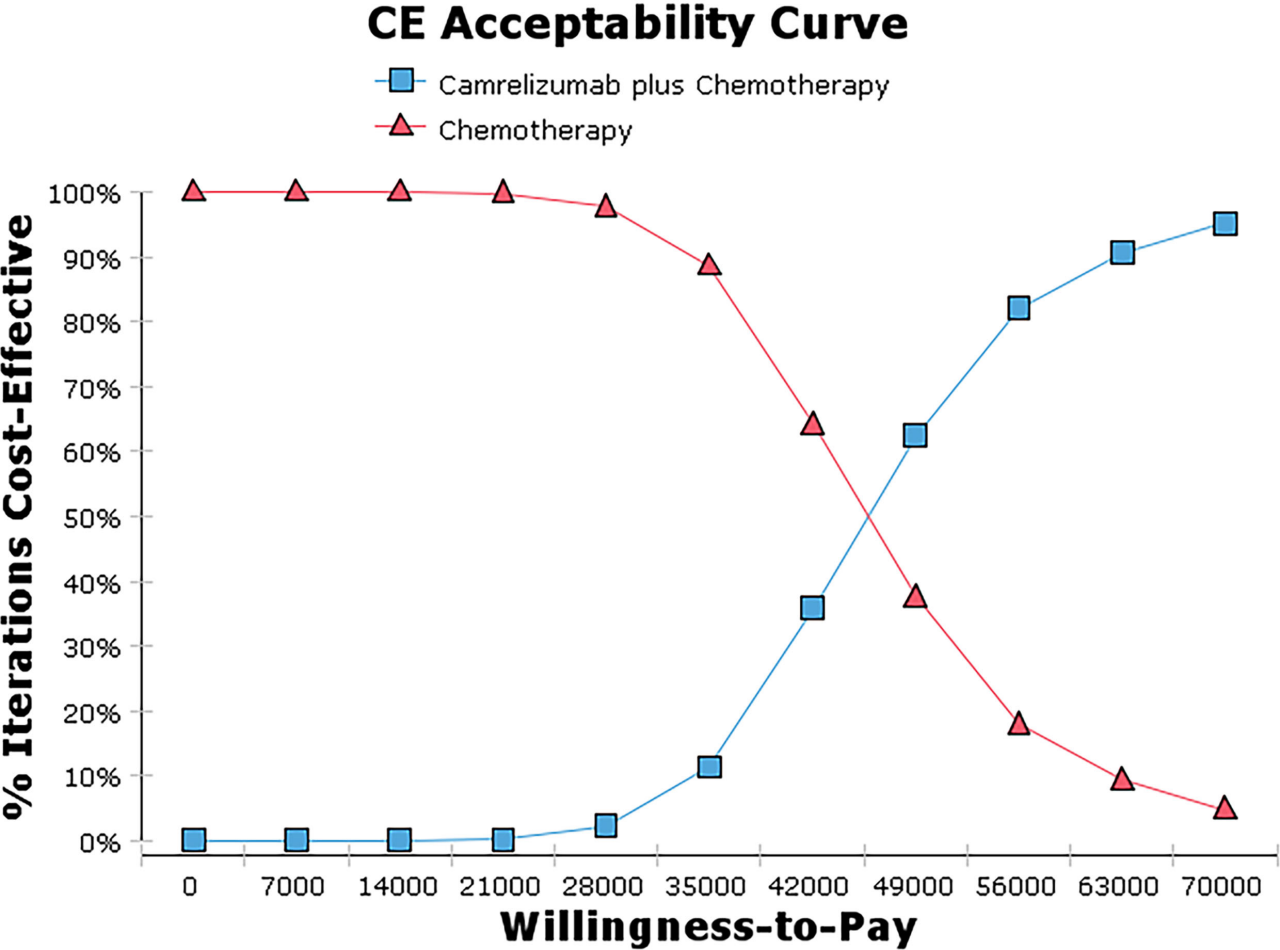
Cost-effectiveness acceptability curve.

The bivalent Monte Carlo simulation was used for probabilistic sensitivity analysis, as shown in **Figures 3**, **4**. The scatter points are in the first quadrant of the coordinate axis, indicating that camrelizumab combined with chemotherapy can result in more QALYs but at a higher cost. As shown in **Figure 4**, when the WTP values were $40,000, $50,000 and $60,000 per QALY, the probabilities of the cost-effectiveness of camrelizumab combined with chemotherapy were 27.1%, 66.7% and 88.0%, respectively.

## Discussion

The main research and development direction of new antitumor drugs in the world is the development of drugs with strong targeting and few side effects. In recent years, tumor immunotherapy represented by ICIs has been the most eye-catching among the many approved new antitumor drugs, in addition to the targeted drugs of various small molecule tyrosine kinase inhibitors and large molecule monoclonal antibodies. Camrelizumab, a PD-1 inhibitor independently developed in China, was approved in June 2020 in combination with pemetrexed and carboplatin for the first-line treatment of unresectable locally advanced or metastatic nonsquamous NSCLC without EGFR and ALK alterations and was included in the first-line treatment recommendation of NSCLC and treatment guidelines of the Chinese Society of Clinical Oncology. On December 28, 2020, it was officially included in the medical insurance drug list with a price reduction of more than 80%. So far, the economic evaluations of camrelizumab have been inconsistent (10, 11), in order to determine the economic efficiency, we established a Markov model to evaluate the two treatment schemes from the aspects of cost and effectiveness.

The results of the basic analysis showed that compared with chemotherapy alone, the ICER of camrelizumab combined with chemotherapy was $43,275.43 per QALY, which was higher than the WTP of $31,510.57 and meant that camrelizumab combined with chemotherapy was not economic. This is different from the previous study which established a partitioned survival model (PSM) for patients and found that the ICER of camrelizumab plus chemotherapy compared with chemotherapy was $-7,382.72 per QALY (10). This may be related to the structural assumption adopted by PSM that the survival functions (i.e. PFS and OS) is independent, resulting in inaccurate predictions of PFS and OS. We established a Markov model which is classic in the economic evaluation of antitumor drugs. Our results are consistent with that of another study based on the same clinical trial (11). And the conclusion is consistent with the systematic review, which showed that ICIs as a first-line treatment for NSCLC has no economic advantage versus chemotherapy regardless of PD-L1 level, but they may be economic in developed countries (12). Of course, the WTP varies from country to country. In European and American countries, the WTP for oncology drugs is significantly higher than that for general drugs and can reach $180,000.

One-Way sensitivity analysis showed that all parameters within the floating range would not reverse the result and the utility value of PFS had the greatest influence on the ICER. The cost of subsequent second-line therapy in the two group also had a significant impact on the results which might be related to the longer duration of the progressive state in terms of the OS of patients. The probability sensitivity analysis results showed that when the WTP values were $40,000, $50,000 and $60,000 per QALY, the probabilities of the cost-effectiveness of camrelizumab combined with chemotherapy were 27.1%, 66.7% and 88.0%, respectively. Considering the uneven regional economic development levels in China, if the WTP is set separately based on the per capita GDP of each province in 2020, then camrelizumab plus chemotherapy can be economical compared with chemotherapy alone in economically developed areas, including Beijing, Shanghai, Jiangsu, Fujian, Tianjin and Zhejiang (29). In addition, the survival time of patients in the final stages of life is more valuable to the patients themselves and society. Therefore, the choice of threshold is also an important factor affecting the economy of the result. It can be questioned whether accepting a higher ICER should be considered in antitumor drugs.

In the CameL study, the PFS times of PD-1-positive patients in the camrelizumab plus chemotherapy group and the chemotherapy alone group were 15.4 and 9.9 months, respectively (HR=0.56, 95% CI 0.39-0.82, one-sided P =0.0011). As of January 2021, the OS was 23.7 months in the chemotherapy group and not yet mature in the camrelizumab plus chemotherapy group. The better survival benefit of camrelizumab in PD-1-positive patients than in all patients may increase the probability of the cost-effectiveness of camrelizumab plus chemotherapy, and further studies may be conducted to determine whether PD-1 testing can help patients choose more cost-effective treatment options.

There are some limitations to this study. First, the survival data were derived from a phase III clinical trial rather than from a prospective real-world study. Second, according to the individual patient, the follow-up treatment plan is different in reality. Third, the utility values of this study were derived from a study of the utility values of NSCLC in the Chinese population, but the difference in the utility values caused by different treatment methods was not reflected. However, the one-way sensitivity analysis results of this study showed that the utility values in the PFS states had the most significant impact on the ICER results. Therefore, it is necessary to refine and measure the effectiveness value of different treatment methods and different states in Chinese patients with NSCLC in the future.

This study may be biased when it comes to real-world decision-making, we look forward to combining the Chinese economic development level with the social average WTP and starting real-world trials of NSCLC as soon as possible to improve the accessibility of new drugs to help cancer patients in the future.

## Conclusions

From the perspective of the Chinese health care system, the camrelizumab plus standard chemotherapy regimen was unable to be cost-effective compared with the standard chemotherapy regimen alone as a first-line treatment for advanced NSCLC if three times the GDP per capita in China is taken as the WTP threshold. However, if the WTP is set separately based on the per capita GDP of each province in 2020, the camrelizumab plus standard chemotherapy regimen can be economical compared with chemotherapy alone in economically developed areas, including Beijing, Shanghai, Jiangsu, Fujian, Tianjin and Zhejiang.

## Data Availability Statement

The original contributions presented in the study are included in the article/supplementary material. Further inquiries can be directed to the corresponding author.

## Author Contributions

TC, HC and LY made substantial contributions to conception and design of the study. TC and QZ performed the data extraction, statistical analysis and drafted the article. RX revised manuscript of important intellectual content. LY reviewed and edited the manuscript. All authors contributed to the article and approved the submitted version.

## Funding

This project was supported by Beijing Health Alliance Charitable Foundation (grant yxky-TQ20210), and Joint Funds for the innovation of science and Technology, Fujian province (2019Y9040).

## Conflict of Interest

The authors declare that the research was conducted in the absence of any commercial or financial relationships that could be construed as a potential conflict of interest.

## Publisher’s Note

All claims expressed in this article are solely those of the authors and do not necessarily represent those of their affiliated organizations, or those of the publisher, the editors and the reviewers. Any product that may be evaluated in this article, or claim that may be made by its manufacturer, is not guaranteed or endorsed by the publisher.

## References

[1] He J Li N Chen W Wu N Shen H Jiang Y. Guidelines for Screening, Early Diagnosis and Early Treatment of Lung Cancer in China (Beijing, 2021). Chin J Oncol (2021) 43:243–68. doi: 10.11735/j.issn.1004-0242.2021.02.A001 33752304

[2] Shi J Wang L Wu N Li J Hui Z Liu S. Clinical Characteristics and Medical Service Utilization of Lung Cancer in China, 2005-2014: Overall Design and Results From a Multicenter Retrospective Epidemiologic Survey. Lung Cancer (2019) 128:91–100. doi: 10.1016/j.lungcan.2018.11.031 30642458

[3] Zang R Shi J Lerut T Wang L Liu C Brunelli A. Ten-Year Trends of Clinicopathologic Features and Surgical Treatment of Lung Cancer in China. Ann Thorac Surg (2020) 109:389–95. doi: 10.1016/j.athoracsur.2019.08.017 31526778

[4] Ettinger DS Wood DE Aggarwal C Aisner DL Akerley W Bauman JR. NCCN Guidelines Insights: Non-Small Cell Lung Cancer, Version 1. 2020. J Natl Compr Canc Netw (2019) 17:1464–72. doi: 10.6004/jnccn.2019.0059 31805526

[5] Markham A Keam SJ. Camrelizumab: First Global Approval. Drugs (2019) 79:1355–61. doi: 10.1007/s40265-019-01167-0 31313098

[6] Song H Liu X Jiang L Li F Zhang R Wang P. Current Status and Prospects of Camrelizumab, A Humanized Antibody Against Programmed Cell Death Receptor 1. Recent Pat Anticancer Drug Discovery (2021) 16:312–32. doi: 10.2174/1574892816666210208231744 33563158

[7] Wang C Sheng C Ma G Xu D Liu X Wang Y. Population Pharmacokinetics of the Anti-PD-1 Antibody Camrelizumab in Patients With Multiple Tumor Types and Model-Informed Dosing Strategy. Acta Pharmacol Sin (2020) 42:1368–75. doi: 10.1038/s41401-020-00550-y PMC8285417 33154554

[8] Shan Q Wang H Han X Guo J Wang Z. Duration of Immunotherapy in Patients With Advanced Lung Adenocarcinoma With Negative Driver Genes: Case Report and Literature Review. Thorac Cancer (2020) 11:3001–6. doi: 10.1111/1759-7714.13600 PMC7529576 32833320

[9] Zhou C Chen G Huang Y Zhou J Lin L Feng J. Camrelizumab Plus Carboplatin and Pemetrexed Versus Chemotherapy Alone in Chemotherapy-Naive Patients With Advanced non-Squamous non-Small-Cell Lung Cancer (CameL): A Randomised, Open-Label, Multicentre, Phase 3 Trial. Lancet Respir Med (2021) 9:305–14. doi: 10.1016/S2213-2600(20)30365-9 33347829

[10] Zhu C Xing X Wu B Liang G Han G Lin C. Cost-Effectiveness Analysis of Camrelizumab Plus Chemotherapy vs. Chemotherapy Alone as the First-Line Treatment in Patients With IIIB-IV Non-Squamous Non-Small Cell Lung Cancer (NSCLC) Without EGFR and ALK Alteration From a Perspective of Health-Care System in China. Front Pharmacol (2021) 24:735536. doi: 10.3389/fphar.2021.735536 PMC8740086 35002693

[11] Xiang G Gu L Chen X Wang F Chen B Zhao J. Economic Evaluation of First-Line Camrelizumab for Advanced Non-Small-Cell Lung Cancer in China. Front Public Health (2021) 10:2021.743558. doi: 10.3389/fpubh.2021.743558 PMC8702426 34957008

[12] Li N Zheng H Zheng B Chen C Cai H Liu M. Economic Evaluations of Immune Checkpoint Inhibitors for Patients With Non-Small Cell Lung Cancer: A Systematic Review. Cancer Manag Res (2020) 12:4503–18. doi: 10.2147/CMAR.S248020 PMC7297344 32606944

[13] Aziz M Tan LE Tan W Toh CK Loke LPY Pearce F. Cost-Effectiveness Analysis of Pembrolizumab Monotherapy Versus Chemotherapy for Previously Untreated Advanced non-Small Cell Lung Cancer. J Med Econ (2020) 23:952–60. doi: 10.1080/13696998.2020.1775620 32462958

[14] Ding H Xin W Tong Y Sun J Xu G Ye Z. Cost Effectiveness of Immune Checkpoint Inhibitors for Treatment of Non-Small Cell Lung Cancer: A Systematic Review. PLos One (2020) 15:e238536. doi: 10.1371/journal.pone.0238536 PMC7467260 32877435

[15] Cai H Zhang L Li N Chen S Zheng B Yang J. Cost-Effectiveness of Osimertinib as First-Line Treatment and Sequential Therapy for EGFR Mutation-Positive Non-Small Cell Lung Cancer in China. Clin Ther (2019) 41:280–90. doi: 10.1016/j.clinthera.2018.12.007 30639208

[16] Weng X Luo S Lin S Zhong L Li M Xin R. Cost-Utility Analysis of Pembrolizumab Versus Chemotherapy as First-Line Treatment for Metastatic Non-Small Cell Lung Cancer With Different PD-L1 Expression Levels. Oncol Res (2020) 28:117–25. doi: 10.3727/096504019X15707883083132 PMC7851532 31610828

[17] Liu Q Luo X Peng L Yi L Wan X Zeng X. Nivolumab Versus Docetaxel for Previously Treated Advanced Non-Small Cell Lung Cancer in China: A Cost-Effectiveness Analysis. Clin Drug Investig (2020) 40:129–37. doi: 10.1007/s40261-019-00869-3 PMC6989620 31679121

[18] Olariu E Cadwell K Hancock E Trueman D Chevrou-Severac H. Current Recommendations on the Estimation of Transition Probabilities in Markov Cohort Models for Use in Health Care Decision-Making: A Targeted Literature Review. Clinicoecon Outcomes Res (2017) 1:537–46. doi: 10.2147/CEOR.S135445 PMC5589111 28979151

[19] Nafees B Stafford M Gavriel S Bhalla S Watkins J. Health State Utilities for Non Small Cell Lung Cancer. Health Qual Life Outcomes (2008) 6:84. doi: 10.1186/1477-7525-6-84 18939982 PMC2579282

[20] Nafees B Lloyd AJ Dewilde S Rajan N Lorenzo M. Health State Utilities in non-Small Cell Lung Cancer: An International Study. Asia Pac J Clin Oncol (2017) 13:e195–203. doi: 10.1111/ajco.12477 26990789

[21] Winning Price of Drugs in Each Province . Available at: https://www.yaozh.com (Accessed May 5, 2021).

[22] Wang L Peng L Peng Y Li S Wan X Zeng X. Comparative Analysis Between 2020 Version and 2011 Version on China Guidelines for Pharmacoeconomic Evaluation. China J Pharm Economics (2021) 16:5–8. doi: 10.12010/j.issn.1673-5846.2021.03.001

[23] Report on Nutrition and Chronic Diseases in China (2020). Available at: https://www.nhc.gov.cn (Accessed June 10, 2021).

[24] Wan N Zhang T Hua S Lu Z Ji B Li L. Cost-Effectiveness Analysis of Pembrolizumab Plus Chemotherapy With PD-L1 Test for the First-Line Treatment of NSCLC. Cancer Med (2020) 9:1683–93. doi: 10.1002/cam4.2793 PMC7050096 31945265

[25] Georgieva M Da SNLJ Aguiar PJ De LLGJ Haaland B. Cost-Effectiveness of Pembrolizumab as First-Line Therapy for Advanced non-Small Cell Lung Cancer. Lung Cancer (2018) 124:248–54. doi: 10.1016/j.lungcan.2018.08.018 30268469

[26] Wu B Zhang Q Sun J. Cost-Effectiveness of Nivolumab Plus Ipilimumab as First-Line Therapy in Advanced Renal-Cell Carcinoma. J Immunother Cancer (2018) 6:124. doi: 10.1186/s40425-018-0440-9 30458884 PMC6247499

[27] Shay R Nicklawsky A Gao D Lam ET. A Cost-Effectiveness Analysis of Nivolumab Plus Ipilimumab Versus Pembrolizumab Plus Axitinib and Versus Avelumab Plus Axitinib in First-Line Treatment of Advanced Renal Cell Carcinoma. Clin Genitourin Cancer (2021) 19:370-370.e7. doi: 10.1016/j.clgc.2021.01.009 33674224 PMC9643032

[28] Lin S Luo S Zhong L Lai S Zeng D Rao X. Cost-Effectiveness of Atezolizumab Plus Chemotherapy for Advanced Non-Small-Cell Lung Cancer. Int J Clin Pharm (2020) 42:1175–83. doi: 10.1007/s11096-020-01076-3 32524512

[29] Statistical Communique of the People's Republic of China on the 2020 National Economic and Social Development (Accessed May 7, 2021).

